# Using virtual reality in lumbar puncture training improves students learning experience

**DOI:** 10.1186/s12909-022-03317-7

**Published:** 2022-04-04

**Authors:** Agathe Vrillon, Laurent Gonzales-Marabal, Pierre-François Ceccaldi, Patrick Plaisance, Eric Desrentes, Claire Paquet, Julien Dumurgier

**Affiliations:** 1 Cognitive Neurology Centre, GHU APHP Nord University Hospital Lariboisière Fernand-Widal, Paris, France; 2 Université de Paris, INSERM U1144, Therapeutic Optimization in Neuropsychopharmacology, Paris, France; 3 Université de Paris, Simulation department, iLumens Paris Nord, Medical School, Paris, France; 4 Université de Paris, Inserm U1153, Epidemiology of Ageing and Neurodegenerative diseases, Paris, France

**Keywords:** Medical education, Education technology, Virtual reality, 3D video, Lumbar puncture, Clinical skills

## Abstract

**Background:**

Lumbar puncture (LP) is a commonly performed medical procedure in a wide range of indications. Virtual reality (VR) provides a stimulating, safe and efficient learning environment. We report the design and the evaluation of a three dimensions (3D) video for LP training.

**Methods:**

We recorded a stereoscopic 180-degrees 3D video from two LPs performed in clinical settings in Fernand Widal Lariboisière University Hospital, Paris, France. The video was administered to third-year medical students as well as to a residents and attendings group during LP simulation-based training sessions.

**Results:**

On 168 participants (108 novice third-year medical students, and 60 residents and attendings with prior LP experience), satisfaction after video exposure was high (rated 4.7 ± 0.6 on a 5-point scale). No significant discomfort was reported (comfort score graded 4.5 ± 0.8 on 5). LP-naive students displayed higher satisfaction and perceived benefit than users with prior LP experience (overall, *P* < 0.05). Trainees evaluated favorably the 3D feature and supported the development of similar tutorials for other medical procedures (respectively, 3.9 ± 1.1 and 4.4 ± 0.9 on 5).

**Conclusion:**

We report our experience with a 3D video for LP training. VR support could increase knowledge retention and skill acquisition in association to LP simulation training.

**Supplementary Information:**

The online version contains supplementary material available at 10.1186/s12909-022-03317-7.

## Background

Lumbar puncture (LP) is an important diagnostic and therapeutic procedure performed in various clinical settings [1–3]. LP is classically performed with the palpation method, where the operator palpates the anatomic landmarks around lumbar spines to identify the needle insertion site (usually L3/L4 or L4/L5 intervertebral disc spaces). This can be challenging in certain patients (e.g. overweight, pregnant patients, back deformation, prior spine surgery) and can potentially cause side effects fostered by suboptimal practice [4–6]. Until recently, training usually involved the learning model of ‘see one, do one, teach one’ where a trainee’s first LP attempt occurs in real life in a high-stakes environment. Novice operators stress levels were found to be high before and during the performance of LP compared to subjects with prior LP experience [7]. The stress of the operator was significantly related to patient confidence in the care provider and risk of postdural puncture headache.

Simulation technology using LP simulators has shown efficiency in improving teaching and operator experience in technical gestures and has been developed in LP [8–11]. It was reported to improve students theoretical knowledge and confidence levels in performing LP, and improved the success rate and the autonomy of the students [8]. However, while simulation is becoming central to healthcare education, it requires significantly more resources than traditional education.

Virtual reality (VR) is emerging as a new method of delivering simulation [12–15]. VR requires the use of hardware (virtual reality headset) to create an immersive simulated environment where the participant is provided with first-person learning experience [16, 17]. Among VR technology, 180- or 360-degree videos allow the exploration of a real or artificial three dimensions (3D) environment [18–20]. They were shown to promote increased engagement over standard two dimensions (2D) videos among medical students [20]. 3D videos can provide learners with a close-to-reality experience that promotes learning. In surgery, it could be used to recreate the environment of an operating room and recording procedures through the eyes of the surgeon could provide students with an optimal learning view [18, 21]. 180- or 360-degree videos constitute an accessible form of VR as they require little material to produce and to deliver to the user. Consequently, they are becoming a more common mode of communicating knowledge and information. Nevertheless, there is a limited evidence on the objective educational benefits of the technology, compared to other teaching techniques [22, 23].

We report the design and development of a prototype stereoscopic 180-degree video of LP in clinical settings and its evaluation in a pilot cohort.

## Methods

### Participants

The study was conducted in Université de Paris, Paris, France, from October 2020 to June 2021. We included participants during LP simulation-based training sessions. The first cohort included residents and attendings, who had already performed LP at the time of training. The second cohort included third-year medical students undergoing LP training session in their regular *curriculum*, with no prior LP experience. Each participant was provided a detailed overview of the study and informed consent was obtained.

### Development of the video

**Fig. 1:**
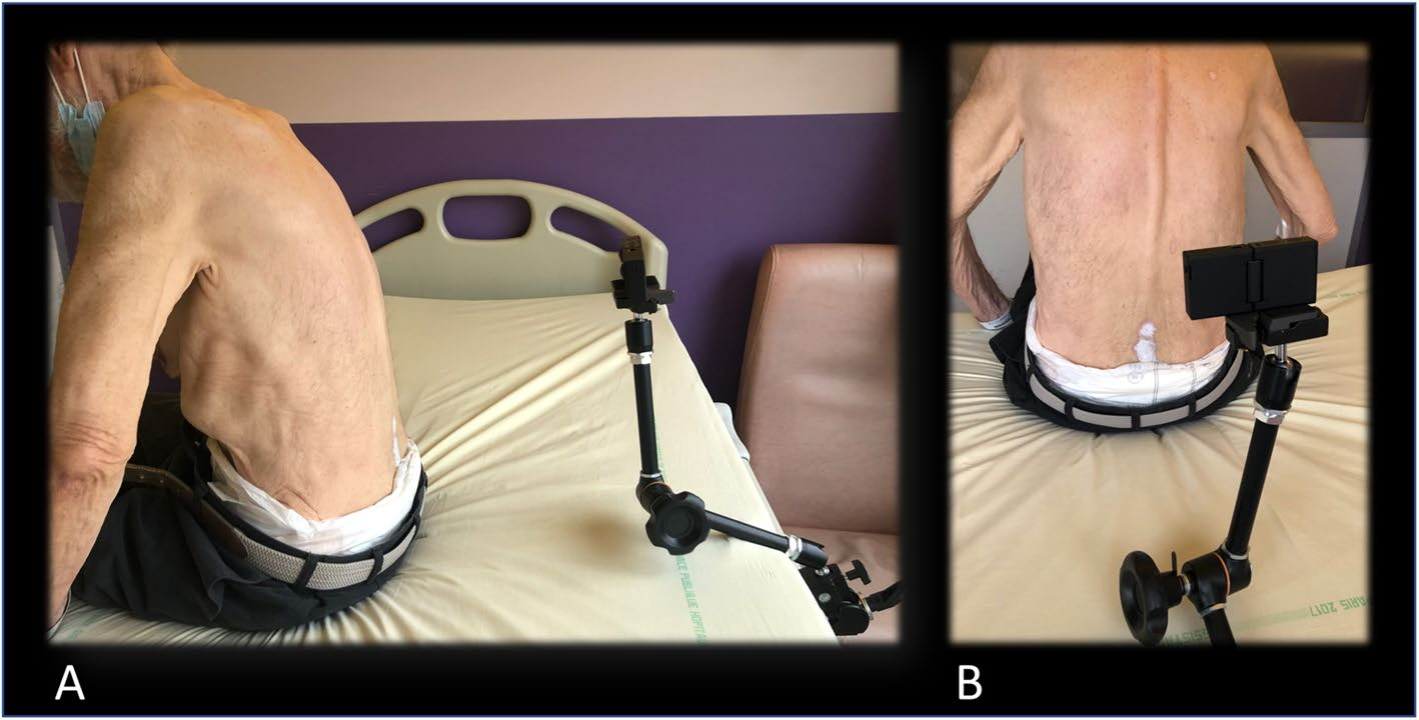
Acquisition of the Video. **A** Lateral view of video acquisition. **B** Operator view of video acquisition

Two LPs performed in clinical settings in the Cognitive Neurology Centre, Lariboisière Fernand Widal Hospital, using atraumatic needle (pencil-point needle) were recorded (Fig. 1). Patients filmed for the video purpose underwent LP in the context of diagnosis work up for neurological symptoms. Procedures recorded included all the steps of the LP procedure: installation of the patients, disinfection steps and sterile conditions setting, placement of the needle, collection of the liquid and end of the procedure. The camera angle gurney attempted to capture the operator view. The video was first recorded in 360 degrees, but the backward view was evaluated as of moderate interest. The video was secondarily recorded in 180 degrees allowing for a stereoscopic video (with distinct right and left eye view): two objectives facing in the same direction each recorded a 180-degree half-sphere image, one for each eye. The camera model used was Insta360 EVO (Arashi Vision Inc., China). The camera was disposed on a swivel arm for optimal filming (Manfrotto, Italy).

Software used for video treatment was: Insta 360 Studio 2019, Insta360 PR plug-in for Première Pro, Adobe Première Pro 2019 and 2020 for editing and Adobe Medias Encoder 2019 and 2020 for encoding and export. No stitching of the images was needed. Captions with explanation on each step of the procedure were added with the text placed in the operator view at zero degree (appendices, Video Script).

**Fig. 2:**
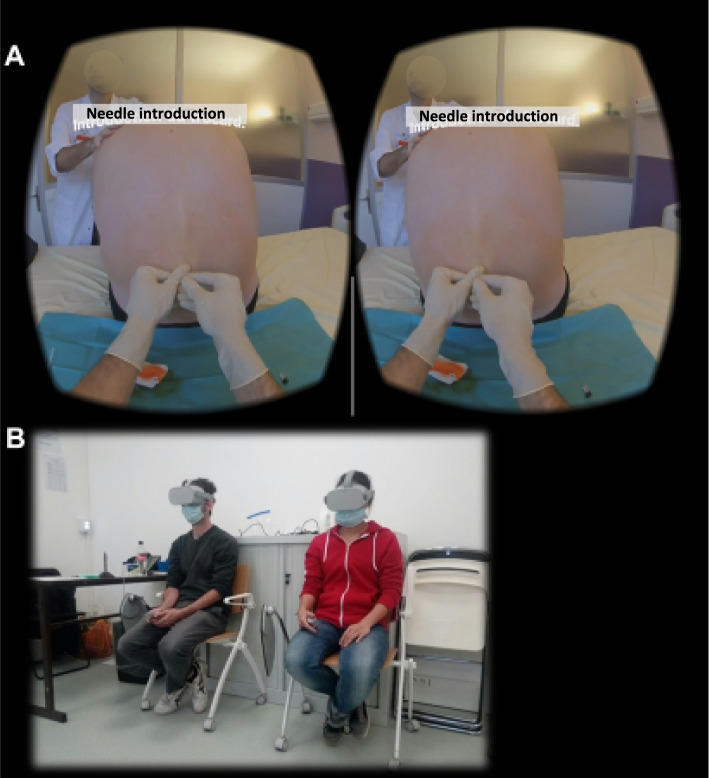
Administration to students of the VR Video. **A** Sample of the stereotaxic view of the video. **B** Two students watching the video with Oculus Go VR headsets

The final video lasted 5 min after editing and was administered through Gizmo VR Video Player, Gizmolite®, Cyprus (Fig. 2). The VR headset used was Oculus Go (Oculus, Microsoft, USA). The video is available on the YouTube platform at the following URL: https://youtu.be/EqtrQUfKO9U.

### Administration of the video

The video was administered once to included subjects during a live 2-h simulation-based training session. Simulation training protocol was previously published [8]. In brief, the course consisted of a rapid evidence-based presentation, reviewing anatomy, indications, complications, and techniques for performing LPs in a regular video format. It was followed by the procedure demonstration and hands-on practice of LPs on simulators (patients lower torso mannequin) with direct feedback by instructors. The video was uploaded on YouTube to allow for repeated viewing, with the option of watching it as a standard video or a 180-degree VR video.

### Evaluation methods

We performed a survey to evaluate comfort during viewing and satisfaction after the administration of the video (Appendices, Survey). Participants were given a questionnaire directly after the LP training session, to assess comfort during viewing, occurrence of any adverse symptoms and perceived benefit and interest of the video. Participants were finally asked to grade their overall satisfaction. Users were also encouraged to provide verbal constructive feedback.

### Statistical analysis

Analysis was performed using GraphPad Prism version 9.00 (GraphPad Software, California USA). Continuous variables were described as mean and standard deviation, median and interquartile range, with indication of data distribution using mode and kurtosis. Categorical variables were described as number of subjects and percentage. Differences between the first cohort (medical student group) and the second cohort (attendings & residents group) were studied using Mann-Whiney test for continuous variables with *Z* test and Chi-squared test for categorical variables. A *p*-value < 0.05 was considered significant with a confidence interval of 95% (CI = 95%).

### Data availability

The datasets analyzed during the current study are available from the corresponding author on a reasonable request.

## Results

One hundred sixty-eight subjects were administered the video and completed the survey during simulation training sessions carried out at Université de Paris, on October 26 and 27, 2020 and June the 8, 9 and 11, 2021 (Table 1). The first cohort consisted of 108 third-year medical students. Forty-four percent of participants had already experienced a 360-degree video in VR. Thirteen percent possessed a personal VR headset. The second cohort included sixty subjects, including residents and attendings with prior LP experience. Video could be administered to 20 subjects by training session of 2 h, using two VR headsets and one dedicated technical engineer. Sanitization of the device and of the administration area was performed between each and every participant.

**Table 1:** Trainees characteristics

***n*** = 168	COHORT 1 Medical students (***n*** = 108)	COHORT 2 Attendings and residents (***n*** = 60)
Age, years, mean (SD)	21.5 (1.7)	27.8 (4.0)
Age, years, median (IQR)	21 (20–22)	26 (25–29)
Men, n (%)	30 (28%)	24 (40%)
Prior exposure to VR, n (%)	48 (44%)	24 (40%)
Possession of VR headset, n (%)	14 (13%)	4 (7%)

Data is presented as mean (SD), median (IQR) or number of subjects (%)

*Abbreviations*: *IQR* Interquartile range, *n* Number of subjects, *LP* Lumbar puncture, *SD* Standard deviation, *VR* Virtual reality

### Comfort of viewing and cybersickness

Comfort score during video exposure was overall high, rated M = 4.5 ± 0.8 on 5 (Table 2). Scores of dizziness, headache and eye discomfort remained low (respectively: M = 0.5 ± 0.9, 0.3 ± 0.8 and 0.3 ± 0.7). Cybersickness was experienced by 5% of subjects. No user had to interrupt the viewing due to adverse symptoms.

**Table 2:** Tolerance of the video

Comfort scale	All subjects (***n*** = 168)
Global comfort score (rated 0 to 5)	M = 4.5 (SD = 0.8) Med = 5 (IQR = 4–5)
Dizziness (rated 0 to 5)	M = 0.5 (SD = 0.9) Med = 0 (IQR = 0–0)
Nausea, n (%)	9 (5%)
Headaches (rated 0 to 5)	M = 0.3 (SD = 0.8) Med = 0 (IQR = 0–0)
Eye pain (rated 0 to 5)	M = 0.3 (SD = 0.7) Med = 0 (IQR = 0–0)

Data is presented as mean M (SD), median Med (IQR) or or number of subjects (%). Parameters were assessed on a 5-point scale (0, no interest, utility or satisfaction to 5, high interest, utility or satisfaction)

*Abbreviations*: *IQR* Interquartile range, *LP* Lumbar puncture, *M* Mean, *Med* Median, *n* Number of subjects, *SD* Standard deviation, *VR* Virtual reality

### Evaluation and interest of the training

The overall level of satisfaction of the included subjects was high: M = 4.7 ± 0.6 rated on a 5-point scale (Table 2). Satisfaction was higher in the medical student group than in the second cohort of subjects with prior LP experience (U [N_medical students group_ = 108, N_Attendings and residents_ = 60] =2598, z = − 2.336, *p* = 0.0037). Analysis of the data showed that the distribution did not differ between groups (medical student group: skewness − 2.94, kurtosis 10.1; attendings and residents group: skewness, − 2.4, kurtosis 10.4).

The interest of the video in addition of simulation training was perceived positively in the overall cohort (M = 4.1 ± 1.0 on a 5-point scale, Table 3). This perceived interest was higher in the first cohort (M = 4.2 ± 0.9) compared to the attendings and residents cohort (3.9 ± 0.9, U [N_medical students group_ = 108, N_Attendings and residents_ = 60] =2641, z = − 1.971, *p* = 0.0338). Analysis of the data revealed that the distribution was negatively skewed and with excessive kurtosis in the medical student group (skewness − 1.16, kurtosis 0;77) whereas the attendings and residents group sample was negatively skewed with negative curtosis (skewness − 0.64; kurtosis − 0.40).

**Table 3:** Evaluation of the video

	All subjects (***n*** = 168)	COHORT 1 Medical students (***n*** = 108)	COHORT 2 Residents and attendings (***n*** = 60)	Mann Withney U	Two samples Z-test	***P***-value*
Satisfaction level (rated 0 to 5)	M = 4.7 (SD ± 0.6) Med = 5.0 (IQR = 5–5) Mode = 5	M = 4.8 (SD ± 0.5) Med = 5.0 (IQR = 5–5) Mode = 5	M = 4.5 (SD ± 0.7) Med = 5.0 (IQR = 4–5) Mode = 5	**2598**	**−2.336**	**0.0037**
Rated interest of VR in addition to simulation (rated 0 to 5)	M = 4.1 (SD ± 1.0) Med = 4.0 (IQR = 4–5) Mode = 5	M = 4.2 (SD ± 0.9) Med = 4.5 (IQR = 4–5) Mode = 4	M = 3.9 (SD = 0.9) Med = 4.0 (IQR = 3–5) Mode = 5	**2641**	**−1.971**	**0.0338**
Rated interest of VR to 2D video (rated 0 to 5)	M = 3.9 (SD ± 1.1) Med = 4.0 (IQR = 3–4) Mode = 4	M = 4.0 (SD ± 1.0) Med = 4.0 (IQR = 4–5) Mode = 4	M = 3.7 (SD ± 1.2) Med = 4.0 (IQR = 3–5) Mode = 4	2727	−1.952	0.0733
Rated interest of extension of VR for other procedures (rated 0 to 5)	M = 4.4 (SD = 0.9) Med = 5.0 (IQR = 4–5) Mode = 5	M = 4.5 (SD ± 0.9) Med = 5.0 (IQR = 4–5) Mode = 5	M = 4.4 (SD ± 0.9) Med = 5.0 (IQR = 4–5) Mode = 5	3066	−0.437	0.4956
Interested in having the video in open-access, n (%)	122 (73%)	91 (84%)	33 (55%)	**─**	**─**	**< 0.0001**^**#**^

Data is presented as mean M (±SD), median Med (IQR) and mode, or n, number of subjects (%). All parameters were assessed on a 5-point scale (0, no interest, utility or satisfaction to 5, high interest, utility or satisfaction)

* LP-naïve medical students versus attending and residents group with prior LP experience, using Mann-Whitney test and Z-test

^**#**^Proportions of participants interested in having the video in open-access were compared using chi-squared

*Abbreviations*: *IQR* Interquartile range, *LP* Lumbar puncture, *M* Mean, *Med* Median, *n* Number of subjects, *SD* Standard deviation, *VR* Virtual reality

The added value of the 3D 180-degree characteristic of video compared to a 2D video was quoted to M = 3.9 ± 1.1 on a 5-point scale and did not differ between groups (U [N _medical students group_ = 108, N _attendings and residents_ = 60] =2727, z = − 1.952, *p* = 0.0733). In both groups, the distribution was negatively skewed and with excessive kurtosis (medical student group: skewness − 1.17, kurtosis 1.29; attendings and residents group: skewness - 1.23, kurtosis 1.50).

Participants believed that the extension of VR to other medical procedures would be of high interest with a mean score of 4.4 ± 0.9, with no difference between groups (U [N _medical students group_ = 108, N _attendings and residents_ = 60] =3066, z = − 0.437, *p* = 0.4956). Distribution was negatively skewed and with excessive kurtosis in both sample (medical student group: skewness − 2.29, kurtosis 6.00; attendings and residents group: skewness − 1.21, kurtosis 0.35).

In addition, 73% of total participants were interested in being able to have the video in open access for repeated viewing. This interest was higher in the medical students cohort (84% versus 55%, chi-squared test, *P* < 0.0001).

## Discussion

In this study, we report the development of a 180-degree stereoscopic LP video in clinical settings. The evaluation of this video in a pilot study on 168 subjects, including 108 LP naive – trainees, showed a good tolerance and feasibility and high satisfaction among the students. Third-year medical students rated higher the perceived benefits and interests of the experience than subjects with prior LP experience, suggesting a real added value of the video. Overall, it was found that implementing 180-degree VR video into LP training could provide a beneficial learning experience.

Previous studies have highlighted the promise of VR video in medical education [14, 17, 24, 25]. Interactive media and online materials provide engaging experience and can help in conceptualizing intricate 3D data (in surgery or anatomy) or integrating the sequences of technical medical procedures [12, 13, 20]. In a survey by Sultan et al., 93% of 169 undergraduate medical students were willing to engage in VR support for medical education [26]. Moreover, knowledge retention and skill acquisition scores were higher after a 360-degree video training compared to a classical lecture in a basic sciences module session. A meta-analysis on 21 studies reported higher accuracy in medical practice by people trained through VR for laparoscopic surgery training in 87% of cases compared to conventional training [27].

LP is an essential tool in daily clinical practice and despite being a relatively safe procedure performed at the patient bedside, a negative attitude appears to persist both in general population and in medical students [4, 28]. In several studies, medical trainees associated LP with a high level of difficulty and a low level of confidence compared to other similar bedside procedures [29, 30]. The development of simulation-based training has allowed practice in a safe environment and significantly increased confidence and real life procedural skills [8, 10].

Our aim was to develop a 180-degree video that would constitute an educational precursor to simulation session. Preliminary feedback received on this new teaching tool was positive. Our preliminary assessment showed that overall satisfaction score was high. We evaluated adverse symptoms including blurred vision, nausea and headache: they were seldom experienced by our participants. Those symptoms, known as cybersickness, are a constellation of symptoms of discomfort and malaise produced by VR exposure, which can limit the use of the medium [31]. Cybersickness has been reported to be frequent in head-mounted display and we believe that the choice of a fixed camera close to the operator view was beneficial regarding tolerance [32]. The video was evaluated as an interesting enhancement to the simulation training by both groups. In the student group, no participant refused the training. Moreover, we had a 100% rate of questionnaire response in students in favor of high engagement in the training. The short length of the video likely allowed participants to maintain their focus and participated to the high satisfaction, as previously reported [19]. However, it is not clear how this high interest would evolve in time if the participants were to increase their familiarity with VR as the majority of participants had no prior 360° video or VR experience. Interestingly, the medical student cohort had a significantly higher satisfaction, perceived a higher interest in the video and were keener to have the video in open access for repeated use than the cohort of attendings and residents with prior LP experience. This suggests that the video provided them with additional content, probably on real-life setting conditions (patient and operator installation during the gesture, communication with the patient during the procedure) that was not given by the mannequin training and that was not perceived by subjects that had already performed LP. A randomized study on 34 medical students showed that training in a simulated setting on endoscopy improved communication skills compared to students who underwent classical theoretical learning [33]. We believe that our tutorial provided insights on interaction and communication with the patient undergoing the LP, thus supporting the learning of medical-related social skills. The 3D characteristic of the video was seen as an addition of moderate interest compared to conventional 2-dimension video. It is known that the perceived interest of 3D increases with the interaction level, lower in a passive video, which could account for this lower rating [14].

Ros et al. developed a 3D LP that increased success in performing LP procedure on a simulator compared to a regular lecture [34]. Students trained with VR displayed reduced latency and made fewer technical errors when performing the LP on the mannequin. Conversely, they performed worse on an oral examination, stressing that the benefit were in precisely and accurately transferring the generalized skill set of the LP on the mannequin. Thus, the immersive tutorial had a specific positive effect on procedural learning, adding evidence on the potential of 3D video. It had also been reported that using a learner’s (first person) perspective in a video better promotes learning of an assembly task, compared to presenting video examples from a third-person perspective [35]. Further elaborating on the concept of Immersive VR Application in the First Person point of view (IVRA-FPV), Ros et al. results on LP training confirm that using first-person point of view improves medical students’ retention of their practical knowledge [34, 36].

Thus, the innovative association of a 3D video providing education about patients installation and full conduct of the procedure in ‘real life’ and of a simulators training allowing for technical skills acquisition that we propose could be an optimal teaching method.

VR 180 – or 360-degree video presents with several advantages. On a technical level, the material requirement is low: a connected screen device (VR headset, computer or mobile device) on which the video has been charged is the only required material. Each group can perform the activity in any environment being provided with the VR headset. A large number of trainees can use this tool simultaneously and its viewing can be repeated as needed. There is no need for continuous supervision. On a practical level, it was easy to produce, compared to immersive VR where the production is time consuming and often requires a large production team. The video could be recorded and edited within a few days. The low cost is also a significant aspect when considering applying it into education especially for low-income settings. Finally, the video modality allows for remote teaching, which is very valuable in the current Covid pandemic context and the associated need to restrict physical contact [36, 37].

We made the choice of a VR 180-degree video [38]. Evolving in a 180-degree space, viewers focus could be guided in a stereoscopic first-person point of view. The video did not provide a full immersion as in 360 degrees or the ability to look in any direction, but in our cases, it was not needed as the patient’s back and the hands of the operator were the points of interest. It has been shown that the use of images recorded from each eye allows for better immersion, characterized by a real sense of ‘presence’ [39]. The 180-degree format also eliminated resolution and bandwidth issues that can be observed with a 360-VR video format.

Our study has several limitations. VR is a relatively new pedagogical technique, so there is no high-quality evidence on the effectiveness of VR – based teaching. Evaluation of the effect of the video training on the performance of LP for the first time on a patient will be the next step of assessment, as it was done for evaluation of simulation training [8]. A better perception of benefit could also be achieved by involving students from several academic levels. Recording different clinical situations (LP procedure on an obese subject or in lateral decubitus) would also allow students to confront themselves to the different situations a physician can encounter in clinical practice [40]. Some of the users encountered cybersickness, a phenomenon that is well described in the literature [41]. Nevertheless as the camera in our video was stationary and contents was of low speed, virtual reality-induced sickness was minimized. All in all, we think our results open a door for future studies to further investigate the development of a VR-based LP training system by demonstrating the feasibility of the administration of the video and encouraging data regarding students’ interest.

## Conclusion

We could develop an innovative VR 180-degree video for LP training. We provide to the community a sample of lumbar puncture VR video that can be freely used for future teaching. In a pilot cohort, the video could be efficiently implemented and its perceived interest and added value to the simulation training was evaluated as high by medical students. A future study will be needed to confirm that our VR video adds value to already established LP simulation training and establish whether it is a valuable tool to improve student learning and LP performance in real-life.

## Supplementary Information

**Additional file 1.**

## Data Availability

Data is available from the corresponding author upon reasonable request.

## Abbreviations

3D: Three dimensions
LP: Lumbar puncture
VR: Virtual reality
SD: Standard deviation
