# Supplementary material for: Using virtual reality in lumbar puncture training improves students learning experience
Source: BMC Med Educ. 2022 Apr 4;22:244. doi: 10.1186/s12909-022-03317-7 (PMC8981937; doi:10.1186/s12909-022-03317-7)
Supplement: Supplementary file 1 — Additional file 1. [file 12909_2022_3317_MOESM1_ESM.pdf]

APPENDICES

Script Lumbar puncture 180-degrees Video

Set-up of the patient.

Doctor 1 "*You will sit on the edge of the bed. You're going to move back like a car backing-up.*"

Flexion of the neck and hips, back as round as possible, to open the intervetebral space as much as possible.

Doctor 1: "*With your hands you will come and hold the bar like this.*"

Patient: "*ok*".

Doctor 1: " *The goal is for you to be relatively comfortable*"

Removal of the anesthetic patch (Lidocain cream).

Patient: "S*o you are drilling in the spine?"*

First disinfection. Use of an alcoholic antiseptic (Chlorexhidine).

Putting on sterile gloves.

Installation of the sterile field. Preparation of the material.

2nd disinfection.

Palpation of the iliac crests. Location of the L4 spine.

Doctor 2 :"*Bend forward please*"

Doctor 1: “ *The head must be bent, the chin towards the chest*”

Doctor 2: “*The back a little bit more forward. That's it. More forward.*”

Doctor 1: “ *To help us, it is necessary to try to round-up the back.*”

Patiente “ *Like a cat?”*

Doctor 1 et 2 : “*Exactly.*”

Doctor 1 “ *Exactly like an angry cat, you see ?*”

Patient and Doctor 1 chatting

Puncture.

Doctor 2 : “*I am going in, Madam.”*

Introduction of introducer

Doctor 3 “ *Here, I am going in.”*

Doctor 2 *“Keep this posture.”*

Introduction of needle. Crossing of the intervertebral ligaments. Penetration into the dural sac. Extraction of obturator from the needle. Verification of CSF reflux.

Collection of the liquid in the tubes.

Doctor 1 “*It can take a few minutes to take the fluid we need. It is drip by drip*.”

10 drips by tubes.

Patient chatting.

Repositioning of the obturator in the needle.

Patient chatting.

Extraction of introducer and needle.

Doctor 2 " *I am cleaning your back* "

Doctor 3 " *I am going to take the needle out*"

Patient " *Yes, do not forget the needle, do not let it in.*"

Doctor 3 " *I am cleaning your back.* "

Application of a bandage.

Patient and Doctor 1 chatting.

Survey on 3D Video - Lumbar puncture in addition to LP simulation training for 3rd year medical students

1. What is your overall level of satisfaction regarding the video ? (On a scale from 0 to 5, 0: unsatisfied, 5 very satisfied)

1 2 3 4 5

2. What was your overall level of confort during video administration ? (On a scale from 0 to 5, 0: unsatisfied, 5 very satisfied)

1 2 3 4 5

3. Did you experience dizziness during video administration ? (On a scale from 0 to 5, 0: unsatisfied, 5 very satisfied)

1 2 3 4 5

4. Did you nausea during video administration ?

YES NO

5. Did you experience dizziness during video administration ? (On a scale from 0 to 5, 0: unsatisfied, 5 very satisfied)

1 2 3 4 5

6. Did you experience headache during video administration ? (On a scale from 0 to 5, 0: unsatisfied, 5 very satisfied)

1 2 3 4 5

7. Did you experience eye pain during video administration ? (On a scale from 0 to 5, 0: unsatisfied, 5 very satisfied)

1 2 3 4 5

8. Is this videao an interresting addition to the LP simulation training ? (On a scale from 0 to 5, 0: no interest, 5 high interest)

1 2 3 4 5

9. Is the video 3D caracteristic of interest compared to classical 2D video ? (On a scale from 0 to 5, 0: no interest, 5 high interest)

1 2 3 4 5

9. Would you like similar videos for other procedures ? (On a scale from 0 to 5, 0: no, 5 yes absolutely)

1 2 3 4 5

10. Would you want open-access to this video for repeated use ? (On a scale from 0 to 5, 0: no, 5 yes absolutely)

1 2 3 4 5

11. Had you seen a 3D video before?

YES NO

12. Do you own 3D headset ? (0: no, 5 yes absolutely)

YES NO

You are:

A Men A Women

What is your age:
